# Oral health behaviour in migrant and non-migrant adults in Germany: the utilization of regular dental check-ups

**DOI:** 10.1186/s12903-017-0377-2

**Published:** 2017-05-19

**Authors:** Fabian Erdsiek, Dorothee Waury, Patrick Brzoska

**Affiliations:** Epidemiology Unit, Institute of Sociology, Faculty of Behavioral and Social Sciences, Chemnitz University of Technology, D-09107 Chemnitz, Germany

**Keywords:** Migrants, Oral health, Dental check-ups, Prevention

## Abstract

**Background:**

Migrants in many European countries including Germany tend to utilize preventive measures less frequently than the majority population. Little is known about the dental health of migrants as well as about their oral health behaviour, particularly in the adult population. The aim of this study was to examine differences in the uptake of annual dental check-ups in adult migrants and non-migrants in Germany.

**Methods:**

We used data from the cross-sectional survey ‘German Health Update 2010’ conducted by the Robert Koch Institute (*n* = 22,050). Data from 21,741 German-speaking respondents with information on the use of dental check-ups was available, of which 3404 (15.7%) were migrants. Multiple logistic regression models were applied to adjust for demographic and socioeconomic confounders, including the place of residence as well as type of health insurance.

**Results:**

Migrants were generally younger, had a lower socioeconomic status and showed a lower utilization of dental check-ups. The unadjusted odds ratio (OR) for utilization was 0.67 (95%-CI = 0.61–0.73). After adjusting for demographic and socioeconomic confounders the chance only increased slightly (adjusted OR = 0.71; 95%-CI = 0.65–0.77).

**Conclusions:**

The analysis shows that migration status is associated with a reduced chance of attending dental check-ups, independently of demographic and socioeconomic factors. The influence of other factors, such as type of health insurance and place of residence had also no influence on the association. Migrants are exposed to different barriers in the health care system, comprising the patient, provider and system level. Further studies need to examine the relevant barriers for the uptake of preventive dental services in order to devise appropriate migrant- sensitive measures of dental prevention.

## Background

Adequate access and utilization of the health system by migrants has become a subject of growing interest in health research given that migrants constitute a large and growing share of the population in many European and other countries. In Germany, migrants account for more than 20% of the population, subsuming both foreign nationals as well as Germans with a migrant background [1]. Migrants differ from the majority populations of the respective host countries in a variety of health aspects comprising the prevalence of certain communicable and non-communicable diseases as well as the utilization of health care services [2, 3].

While the provision of health care services for regular migrants in Germany is nominally equal to that of non-migrants and covered by the statutory or private health insurance (the situation is different for refugees and asylum seekers who only have limited access to health care [4]), the needs and expectations of migrant populations are often not adequately met in health care. This can negatively impact health care utilization and amongst others becomes evident in lower utilization of preventive health care services [3, 5–7]. In Germany, migrants have been found to use cancer screenings, vaccinations and rehabilitative services less often than the majority population [8–10].

In terms of oral health, studies addressing differences between migrants and non-migrants in Germany and other Western European countries, usually focus on oral and dental health of migrant children and/or specific migrant subgroups [11]. These studies mostly conclude that children with a migrant background are more likely to have a poorer oral health, manifesting predominantly in higher prevalence of caries [12–14]. The German Health Interview and Examination survey for Children and Adolescents (KiGGS) has, among other topics, examined demographic and socioeconomic determinants of dental hygiene and preventive behaviour in children and adolescents, concluding that children and adolescents with a migrant background more often did not reach recommended frequencies for tooth brushing and were less likely to use fluoride tablets [15, 16].

An important aspect of maintaining and promoting oral health is the utilization of regular dental check-ups at least once a year [17]. Studies from several countries show that migrants utilize dental check-ups less frequently than the respective autochthonous populations [18–21]. In Germany, research on the utilization of dental prevention measures among migrants is scarce, especially for adults. Small-scale studies have shown that migrant children were less likely to receive fissure sealings [22, 23] and orthodontic treatment [12] than non-migrant children. Results from the KiGGS survey have also shown that utilization of annual dental check-ups is lower among migrant children and adolescents than among their non-migrant counterparts in Germany, even after adjustment for age and socioeconomic status [16]. A recent descriptive analysis shows that the utilization of annual dental check-ups among adult migrants is less common for men and women of all groups of socioeconomic status [24]. The role of other confounding variables such as a private insurance and being married, which are also positively associated with regular dental visits [21, 25–27], was not accounted for. The aim of this paper is to examine whether migrant and non-migrant adults differ in the utilization of annual dental check-ups, taking into account socioeconomic and demographic aspects as well as additional contextual factors.

## Methods

We did a secondary analysis using data from the cross-sectional telephone survey ‘German Health Update 2010’, carried out between September 2009 and July 2010 by the Robert Koch Institute, a scientific institution of the German Federal Ministry of Health [28]. The study included information on health behaviour, health outcomes and utilization of health services. The sample was drawn from the population of adults in Germany aged 18 years or older who lived in a private household with a landline telephone. In order to determine the sample size, a power calculation to identify 50% prevalences with a +/−5% error risk per cell (differentiating between regions, sexes and three age groups) was performed. Sampling was conducted using a modified random-digits approach, resulting in a sample of 12,483 female and 9567 male respondents [29]. Since only German-speaking adults were included, the sample is not representative for migrants with low or no proficiency of the German language. No other inclusion criteria were set.

The outcome of interest was the utilization of dental check-ups in the 12 months prior to the interview as a dichotomous variable. To determine migration status, we used a broader definition: individuals were defined as migrants if they had migrated to Germany themselves or if at least one of their parents had migrated to Germany, following the approach of other studies in the field [9]. To control for potentially confounding factors, we included several variables pertaining to demographic, socioeconomic and health aspects which often differ between migrants and non-migrants and which are associated with the utilization of health services [21, 24–27]. We included information on the respondents’ sex and age (in 10-year age groups), as well as on their socioeconomic status (SES). In our analysis, we used SES as a categorical variable, distinguishing between high, middle and low status, using the measurement and categorization of Lampert et al. [30], which includes information on general and vocational educational, occupational status and net equivalent income. In terms of place of residence, information was included on whether respondents lived in West or East Germany and whether they lived in predominantly urban or more rural areas to take into account regional differences in availability of dental services. Cohabitation status was used with a distinction between respondents living together with a spouse/partner or not. Furthermore, we considered the type of health insurance (statutory as compared to private or other).

We calculated descriptive uni- and bivariate statistics to analyse the sample structure and to compare between groups according to migration status, using chi-square-tests and Wilcoxon-Mann–Whitney tests where appropriate. The association between migration status and use of dental check-ups was assessed by means of multivariable logistic regression modelling [31] adjusted for the aforementioned covariates as potential confounders. All analyses were done using Stata 13 [32].

## Results

Due to missing information on the outcome variable or on one of the predictors, 309 individuals (1.4%) were excluded from analysis. Of the 21,741 respondents remaining, 15.7% had a migrant background. Among the migrant participants, 2474 had either migrated themselves or had two parents who migrated to Germany, while 930 had not migrated to Germany but had one parent with migration experience. Differences between migrants and non-migrants were identified for some, but not all aspects (Table 1). The larger portion of the sample were female (56.7%), with no significant difference between groups. Comparison of the age composition showed that migrants were significantly younger. Around 50% were younger than 40 years, while this amounted to around 31% in the non-migrant group. Migrant respondents also more often had a lower socioeconomic status and were more often insured through statutory health insurance than non-migrants. In terms of place of residence, migrants lived significantly more often in West Germany and in urban areas. In both groups, the majority lived together with their spouse or a partner. With respect to the outcome it can be observed that utilization of dental prevention was overall lower among migrants (72.6%) than non-migrants (79.8%), corresponding to a crude odds ratio (OR) of 0.67.

**Table 1:** Description of the study sample by migrant status (German health update 2010 survey, *n* = 21,741)

Variable		non-migrant *n* (%)	migrant *n* (%)	*p*-value
Sex	male	7947 (43.5)	1447 (42.6)	0.33
	female	10,317 (56.5)	1948 (57.4)	
Age groups	18–29 years	2866 (15.7)	901 (26.5)	<0.01
	30–39 years	2720 (14.9)	784 (23.1)	
	40–49 years	4211 (23.0)	691 (20.4)	
	50–59 years	3407 (18.7)	444 (13.1)	
	60–69 years	2799 (15.3)	361 (10.6)	
	70–79 years	1756 (9.6)	172 (5.1)	
	80+ years	505 (2.8)	42 (1.2)	
Socioeconomic status	high	6278 (34.4)	965 (28.4)	<0.01
	middle	10,205 (55.9)	1827 (53.8)	
	low	1781 (9.8)	603 (17.8)	
Type of health insurance	statutory	14,939 (81.8)	3024 (89.1)	<0.01
	private/other	3325 (18.2)	371 (10.9)	
Place of residence	West Germany	14,664 (80.3)	3034 (89.4)	<0.01
	East Germany	3600 (19.7)	361 (10.6)	
Urban residence	Yes	12,501 (68.4)	2758 (81.3)	<0.01
	No	5763 (31.6)	637 (18.7)	
Living together with spouse/partner	Yes	11,373 (62.2)	2069 (60.9)	0.15
	No	6891 (37.8)	1326 (39.1)	
Use of dental check-up in the last 12 months	Yes	14,579 (79.8)	2463 (72.6)	<0.01
	No	3685 (20.2)	932 (27.4)	

When adjusting for the effects of sex, age, socioeconomic status and other confounders in the second model, the chance for utilization slightly increased, but was still considerably lower than for non-migrants (OR = 0.71) (Table 2). In the adjusted model, the majority of the covariates were significantly associated with the outcome. Apart from the oldest group, all age groups had an increased chance of participation compared to the youngest group ranging from 18 to 29 years. While consistently higher than the reference group, the chance for using dental check-ups peaked for the age group from 40 to 49 years and decreased with higher age groups. Chances for utilization also increased consistently with higher socioeconomic status. Being female and living together with a partner or spouse was also found to enhance the chance of utilizing dental check-ups, while living in West Germany was associated with a reduced chance of utilization.

**Table 2:** Results of the multivariable logistic regression model with utilization of dental check-ups in the 12 months prior to the interview as the dependent variable. Odds ratios (OR) and 95% confidence intervals [95%-CI] (German health update 2010 survey, *n* = 21,741)

Independent variable		Model 2		
		OR	95%-CI	
Migration status (ref.: non-migrant)	migrant	0.71	0.65	0.77
Sex (ref.: male)	female	1.88	1.76	2.00
Age (ref.: 18–29 years)	30–39 years	1.36	1.21	1.52
	40–49 years	1.65	1.48	1.85
	50–59 years	1.43	1.27	1.60
	60–69 years	1.25	1.11	1.41
	70–79 years	1.15	1.01	1.31
	80+ years	0.39	0.32	0.47
Socioeconomic status (ref.: low)	middle	1.70	1.54	1.87
	high	2.36	2.10	2.65
Type of health insurance (ref.: statutory)	private or other	0.78	0.71	0.85
Living together with a partner/spouse (ref.: no)	yes	1.41	1.31	1.51
Place of residence (ref.: East)	West Germany	0.87	0.79	0.95
Urban residence (ref.: no)	yes	0.94	0.87	1.01

## Discussion

The aim of this analysis was to compare the utilization of annual dental check-ups between migrants and non-migrants in Germany. Using a national sample of adults, the study shows that utilization was lower among migrants than non-migrants. Even after adjusting for a variety of individual and structural factors, having a migrant background was still associated with an almost 30% lower chance of utilization. These findings are in line with studies from other countries [18–21] and suggest that additional factors associated with migrant background need to be considered when addressing differences in the utilization of dental prevention.

All of the covariates we included as potential confounders were significantly associated with the outcome, with the sole exception of living in an urban area. The associations identified were generally in line with findings from other studies on the utilization of dental services. Having a higher socioeconomic status was associated with an increased chance for utilizing dental prevention, which is consistent with results from previous research [27, 33]. The lower chance of utilization among adults with private insurance or other type of funding is likely to be related to differences in co-payment and reimbursement agreements [34]. Being female and living with a partner (or being married) was also found to increase the chance of dental service utilization among adults in Denmark [21].

Utilization of health services by migrants can be limited due to a variety of potential barriers on the patient, provider and system level. On the patient level, culturally specific perceptions and beliefs of health and illness, low health literacy (including knowledge about the health system), limited language skills, perceptions and attitudes towards health services and personnel and low family or social support are important factors limiting the utilization of preventive care [35]. Since the sample included only adults with a high proficiency of the German language, language barriers can be excluded as a major influence in our sample. In addition, culturally specific health and illness beliefs may influence the utilization of preventive measures [35].

Some migrants have been reported to not using health services until they exhibit symptoms [36]. This means they probably do not perceive the necessity or benefits of prevention as strongly as non-migrants and cannot be reached to the same extent by existing preventive efforts. Low health literacy or a lack of information on the German health system may further reduce the chances of using preventive services. Studies have shown that one of the main risk factors for caries among migrant children in Germany is parents’ lack of knowledge and awareness of the negative effects of sugar and sweet foods [37]. Migrant parents also tend to underestimate the benefits of prophylactic efforts and products (e.g. fluoride tablets) [16]. Although these studies surveyed parents on the oral health of their children, the findings probably may also be attributable to their perceptions of oral health in general. This, however, needs to be verified by future studies examining knowledge and beliefs of adults concerning their own oral health.

Acquisition of information on the structure of the German health system, available preventive services and financial aspects of healthcare can be problematic as well. A considerable barrier to accessing health care including measures of prevention is the cost of these services [9]. In Germany, dental check-ups once every 6 months are exempt from co-payment. If this is unknown, anticipated cost may still be a considerable barrier.

Also communication problems can be a source of underutilization, despite high language proficiency. Different modes or patterns of communication may negatively influence the relationship between dentists and migrant patients and through this impede health service utilization. A study among parents in Germany found that, compared to German parents, Turkish parents reported receiving insufficient information more often, trusted their dentist less and felt more often that their dentist was trying to give them a guilty conscience if the children had poor dental hygiene [38].

Barriers on the provider and system level comprise, amongst others, a limited cultural sensitivity of health services, missing information on existing services, a largely biomedical understanding of health and negative perception of or attitudes towards migrant patient groups including discrimination [35]. While different barriers have been identified that migrants in Germany encounter in different sectors of the health care setting [2, 36, 39], the relevant barriers responsible for the underutilization of dental check-ups yet need to be identified.

Some limitations of our study need to be considered. The sample is not representative for the general population of migrants in Germany. This is reflected in the fact that high proficiency of the German language was an inclusion criterion and in the fact that migrants were underrepresented in the sample [28]. This limits generalizability of our findings. Given the positive selection and the known effect of poor language proficiency, the observed differences between migrants and the majority population are likely underestimated. Another limitation is the categorization of the migration status. The term migrant comprises a very heterogeneous group with a large diversity in terms of religious beliefs, culturally specific perceptions, values, languages and length of stay. As these factors may also be associated with the utilization of preventive measures of dental care [40, 41], further research should examine how they are related to the lower utilization observed for migrants in Germany. This could inform the development of targeted interventions. Although we could not take these diversity factors into account, we were able to include not only migrants with a non-German nationality but, unlike other health research studies in Germany which use secondary data, to also consider Germans with a migrant background [2]. Around 7% of the German adult population are estimated to have no landline telephone connection [42]. Due to the design of the ‘German Health Update’ survey, these individuals were not included in the sample. However, considering the low number of households that are mobile-only or without any phone, we consider the resulting bias to be small. Furthermore, official statistics show that the socioeconomic composition of the sample is very similar to that of the population in Germany [43].

## Conclusions

The utilization of annual dental check-ups in Germany is lower among adult migrants than among non-migrants, even after taking into account several demographic, socioeconomic and contextual factors. These findings are in line with findings from other studies on the utilization of preventive dental care measures among migrant children as well as studies on other types of preventive services. Our findings highlight the necessity of culturally sensitive and targeted services in the dental care setting. This includes communication on the system level (in terms of providing information on existing services and structures) as well as on the provider level (in terms of patient-provider interaction), the design and delivery of services (in terms of patient approach, waiting times and opening hours) as well as patient education to enhance health literacy. Future studies, applying qualitative or mixed-method approaches, need to explore the relevant barriers which migrants encounter in preventive dental care.

## Abbreviations

95%-CI: 95%-confidence interval
KiGGS: German Health Interview and Examination survey for Children and Adolescents [Studie zur Gesundheit von Kindern und Jugendlichen in Deutschland]
OR: Odds ratio
SES: Socioeconomic status
